# Localized Subcutaneous Insulin-Derived Amyloidosis Excised after Evaluation Using Ultrasonography in a Patient with Type 2 Diabetes Mellitus

**DOI:** 10.1155/2017/3985214

**Published:** 2017-12-18

**Authors:** Seiya Hagiwara, Shinji Taneda, Takaya Fukumoto, Kazuya Hagiwara, Minoru Kikuchi, Tetsunori Kimura, Hidetaka Nakayama, Naoki Manda

**Affiliations:** ^1^Manda Memorial Hospital Diabetes Center, Sapporo, Japan; ^2^Fukumoto Dermatopathology Clinic, Nara, Japan; ^3^Yamahana Dermatology Clinic, Sapporo, Japan; ^4^Sapporo Dermatopathology Institute, Sapporo, Japan

## Abstract

A 62-year-old man with type 2 diabetes mellitus, who had been on insulin therapy for the past 20 years, was found to have subcutaneous mass formation in the abdomen during a workup of worsened glycemic control. Because of suspected amyloid deposition, he was advised to avoid injections to the mass, which led to improvement of glycemic control. However, he strongly requested mass excision and was hospitalized. After evaluation using ultrasonography and computed tomography, a total mass excision was performed, and a diagnosis of insulin-derived amyloidosis was made. Comparison of the ultrasonographic and histopathological findings demonstrated that the location of the amyloid deposition nearly corresponded to the hypoechoic region. This case highlights that ultrasonography, which is a noninvasive imaging modality, can be useful for detection of insulin-derived amyloidosis.

## 1. Introduction

Insulin-derived amyloidosis (IDA) has been described as a cutaneous complication occurring at insulin injection sites and is known to cause impaired absorption of insulin [1–8]. We here report a case of subcutaneous mass formation in a type 2 diabetes mellitus patient, in whom a total mass excision was performed after evaluation using an insulin tolerance test and imaging, along with discussion on characterization of amyloid deposition primarily with comparison of ultrasonographic and histopathological findings.

## 2. Case Report

**Figure 1:**
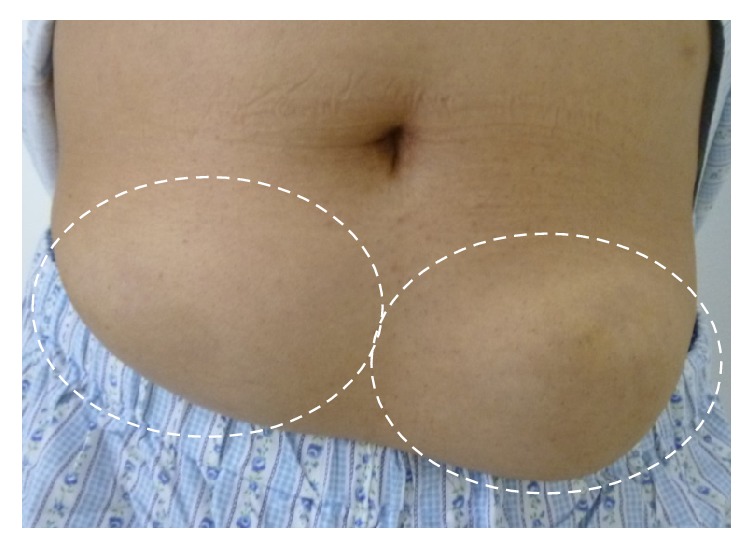
Physical examination of the affected area: two subcutaneous masses were present, each measuring 50 mm, in the left and right subumbilical regions, with minor subcutaneous hemorrhage and tenderness (indicated by broken-lined ovals).

**Figure 2:**
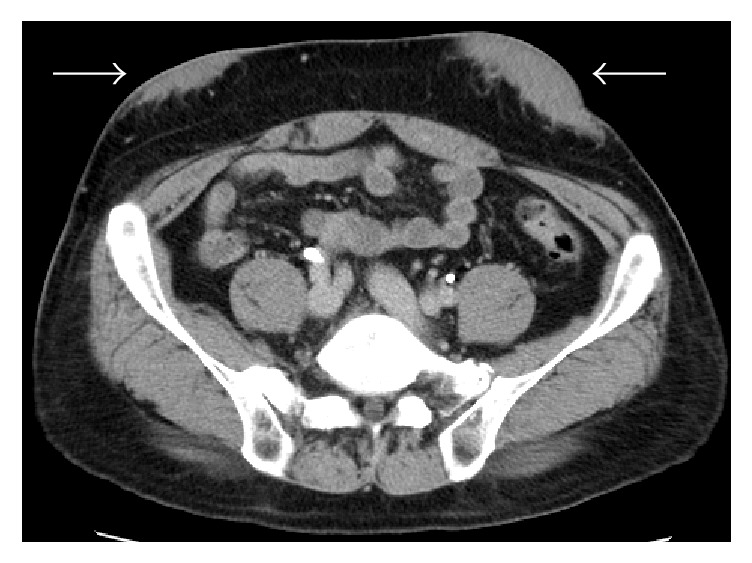
Contrast-enhanced CT of the abdomen (equilibrium phase): in the left and right subumbilical regions, subcutaneous masses with irregular margins are observed, each measuring approximately 60 mm. The density is heterogeneous and higher than that of the surrounding adipose tissue, without contrast-enhancement effect (indicated by arrows).

**Figure 3:**
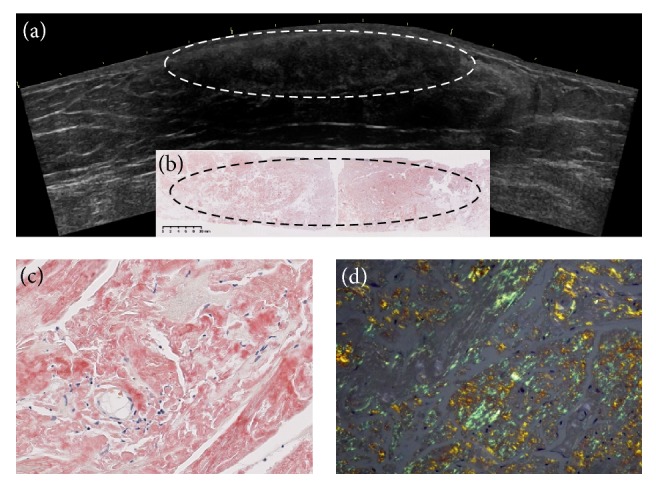
Comparison of preoperative abdominal ultrasound findings (a) and Congo red-stained histopathological findings (b). The high magnification figures (×200) of Congo red staining (c) and its green birefringence (d). (a) On ultrasonography, the subcutaneous mass is shown as a somewhat heterogeneous, hypoechoic area under the dermis. (b) Congo red-stained section of the mass shows diffuse, irregular, lumpy amyloid deposition (indicated by broken-lined ovals). When the material was stained with Congo red staining (c) and seen with polarized light, we saw green birefringence (d), diagnostic of amyloid.

62-year-old man with diabetes mellitus was diagnosed in 1984, treated with four injections per day of insulin since 1994. Outpatient treatment at Manda Memorial Hospital was started in 2004. In May 2011 his HbA1c was 6.7% (National Glycohemoglobin Standardization Program) and stable, but subsequently his blood glucose levels gradually increased, requiring gradual increase in the insulin dosage. In December 2012, his HbA1c worsened to 8.3%, and the total daily dose of insulin was increased to 79 units (lispro 39 units and glargine 40 units). In January 2013, injection site assessment revealed palpable subcutaneous masses in the left and right subumbilical regions. An interview at this point revealed that he had noticed the presence of them since approximately half a year earlier. He was advised to rotate the injection sites, which led to frequent occurrence of hypoglycemia. Thus, the total daily dose of insulin was then reduced by 41 units to 38 units (lispro 22 units and glargine 16 units). In July 2014, his HbA1c improved to 5.8%. These masses were strongly suggested to be IDA, for which follow-up observation was initially advised. In September 2014, however, he had worsening of subcutaneous hemorrhage on the surfaces of the masses and spontaneous pain due to pressure from a trousers belt. Thus, in accordance with his strong request, in November 2014 he was admitted to this hospital for detailed examination and mass resection. Upon admission, laboratory test values showed no particular abnormalities (i.e., fasting plasma glucose 98 mg/dL, HbA1c 5.8%, fasting C-peptide immunoreactivity 1.01 ng/mL, C-reactive protein 0.03 mg/dL, glutamic acid decarboxylase antibody < 0.4 U/mL, insulin antibody < 125 U/mL, nonspecific immunoglobulin E (IgE) 79 IU/mL, and human insulin specific IgE < 0.1 UA/mL). The body mass index was 23.9 kg/m^2^ (body weight, 57.5 kg). Physical examination of the affected area revealed two movable, hard, subcutaneous masses, each measuring 50 mm, in the left and right subumbilical regions. The masses had smooth surfaces, partly with subcutaneous hemorrhage and tenderness (Figure 1). Contrast-enhanced CT of the abdomen (Figure 2) demonstrated subcutaneous masses with irregular margins, each measuring approximately 60 mm, in the left and right subumbilical regions, with the density higher than that of the surrounding adipose tissue, and without contrast-enhancement effect. Ultrasonography showed a poorly defined heterogeneous hypoechoic mass, measuring 66 (height) × 85 (width) × 20 mm (depth), in the subcutaneous fat layer in the left subumbilical region (Figure 3(a)) and another mass with similar findings on the other side as well. Then, for assessment of impairment of insulin absorption at the left subcutaneous mass site, an insulin tolerance test (data not shown) with lispro 9 units injection followed by intake of a 500 kcal breakfast was performed. At the mass site, compared with nonaffected regions, the peak blood glucose level was 2.23 times higher and the peak blood insulin level was 0.77 times higher, while the AUC_0–3 hr_ value for blood glucose was 2.02 times higher and the AUC_0–3 hr_ value for blood insulin was 0.69 times higher. These data confirmed marked impairment of insulin absorption at the mass site.

Total excision of the left subcutaneous mass under local anesthesia was finally decided with the consent of the patient. After ultrasonographic assessment to determine the resection margin, under local anesthesia it was completely excised along with the adjacent epidermis and subcutaneous fat layer. The histopathological picture (Congo red staining) is shown in Figure 3(b), with the high magnification figures of Congo red staining (Figure 3(c)) and its green birefringence (Figure 3(d)). On hematoxylin and eosin staining, the epidermis and the dermis showed no particular changes, but the subcutaneous fat tissue showed a large amount of lumpy deposition of a homogenous eosinophilic material that was irregular in shape. The material was positive for Congo red staining and direct fast scarlet staining, while polarized light microscopy showed green birefringence. These findings were compatible with amyloid. Azan staining showed deposition of collagen fibers. Immunohistochemical staining was negative for beta-2-macroglobulin, negative for AA-amyloid, and positive for insulin. These findings indicated deposition of insulin-derived amyloid, partly accompanied by collagen fibers. Comparison of the ultrasonographic image of the subcutaneous mass and the Congo red-stained section demonstrated that the location of the diffuse, irregular, lumpy, Congo red-positive amyloid deposition nearly corresponded to the somewhat heterogeneous hypoechoic region under the dermis.

## 3. Discussion

The diagnosis of IDA is made based on clinical evidence of a palpable subcutaneous mass and histopathological evidence of amyloid deposition [8]. Imaging investigation by plain CT typically shows a heterogeneous mass with the density higher than that of the surrounding adipose tissue. To our knowledge, this is the first case report of IDA in which histopathologic examination of the total excised mass demonstrated amyloid deposition nearly corresponding to the hypoechoic region after ultrasonography was used to determine the presence of mass formation. Only a few previous reports of ultrasonographic findings of IDA have been found [9, 10]. We have recently reported the feasibility of ultrasound diagnosis of IDA [11]. In addition to the ultrasound detectability and findings, we investigated insulin absorption rate, insulin dosage, and HbA1c before and after shifting the insulin injection site in 22 IDAs including this case. The detectability of IDAs on ultrasound was 100%, in which 59.1% were palpable lumps and 40.9% was not palpable. The palpable type showed lower echo intensity and was harder than the nonpalpable type. The blood flow decreased in IDA, especially in the palpable type. IDA had a low insulin absorption rate, especially in the palpable type. HbA1c and insulin dosage decreased after shifting the injection site. The palpable type had more insulin reduction than the nonpalpable. This case was the palpable type and had a low insulin absorption rate, too.

With regard to relevant cases assessed using ultrasonography, Perciun reported a patient treated with insulin for 34 years who had a hypertrophic area at a subumbilical injection site [12]. Ultrasonography showed a hypoechoic area in the subcutaneous tissue at a site of frequent insulin injections, and change of injection sites led to reduction of the daily insulin dose from 205 units to 70 units. Although the authors suspected a “posthaemorrhagic fat necrosis process” without histopathological assessment, the findings of the case could be amyloidosis, considering amyloidosis was hypoechoic as we have already reported. There has been no report on the differential diagnosis of subcutaneous lesions by ultrasonographic findings between IDA and other subcutaneous indurations such as lipohypertrophy and allergic reaction. IDA was presented as a low echoic and elastic hard mass and the blood flow was low by an ultrasonography [11]. These findings may be useful for the differential diagnosis from lipohypertrophy or allergic reaction. Further accumulation and examination of cases should be necessary for the differential diagnosis. In patients on insulin therapy, assessment for possible IDA is warranted, where ultrasonography, a noninvasive and simple imaging modality, can be useful for detection of the disease.
